# Substance of a Memoir Lately Published by Cit. Chaussier, Professor in the School of Medicine, at Paris, on the Means of Preserving the Dead Bodies of Animals from Putrefaction, and Preserving Their Essential Form, and Even by Giving Them Freshness and the Appearance of Life

**Published:** 1804-04-01

**Authors:** 


### Substance of a Memoir *lately published by Cit.* Chaussier, *Professor in the School of Medicine, at Paris, on the* Means *of preserving the* dead Bodies *of* Animals *from* Putrefaction, *and preserving their essential* Form, *and even by giving them* Freshness *and the* Appearance *of* Life.

The bodies of animals, when they are deprived of life, exposed to the action of the atmosphere, plunged in water, or buried in the earth, speedily pass to a state of putrefaction, and become the food of worms and insects, and after a time, always very short, the mass of their flesh becomes reduced to some hectograms of a dust, which the winds disperse, which the waters carry away, and which vegetables absorb for their nourishment. This destruction, this alteration, so great, and so rapid, is a necessary consequence of the quality, of the very nature of their constituent parts, of their tendency to decomposition, and of the considerable quantity of fluids which they contain in comparison with the solids. In order, therefore, to preserve the carcases of animals, or any of their parts, we must necessarily change the natural order of their composition, and by the help of different agents, determine new combinations, which, by preserving the essential form and texture, may be, at the same time, imputrescible, unalterable by the vicissitudes of the atmosphere, and unattackable by insects.

After these primary considerations, which serve as a basis to his researches, Cit. Chaussier examines the different processes which have been successively employed, for the preservation of entire carcases, or of anatomical pieces; and after having remarked that some are illusory, and that the others do not protect animal substances from the voracity of insects; that all are attended with the inconvenience of altering the essential configuration, and of reducing the body to a shapeless mass, he announces the solution of sur-oxygenated muriat of mercury, in distilled water, as the most likely method to accomplish the desirable object. The use of this saline solution on animal substances, must vary according to the size and the condition of the object which it is intended to preserve. If it be only a separate piece, like the most of anatomical preparations, it is sufficient to plunge it in a solution of sur-oxygenated muriat of mercury, and to add in the vase one or more knotted parcels of fine linen, which contain some some grammes of this mercurial salt, a precaution essential to the ensuring its remaining always equally saturated. After ten, twenty, or thirty days of immersion, that is to say, when the part has been penetrated through its whole extent by the saline solution, when a new combination has been operated through all its points, we may draw it out of the liquor and place it in a narrow-necked jar or bottle, filled with distilled water, lightly charged with sur-oxygenated muriat of mercury, or else it may be exposed in a well-aired place, sheltered from the sun and from dust; it will then get dry, by little and little acquire a consistence and a hardness resembling wood; and in this state, says the Professor, it can no longer be either altered by the air, or attacked by insects. This, indeed, has been sufficiently proved by his experiments; Cit. Chaussier having for many years abandoned pieces thus prepared to insects and to the vicissitudes of the atmosphere.

The preservation of the entire body requires particular care and attention, all the details of which it would be impossible to comprise in a simple notice. It is in some measure a new art, the process of which can only be well executed by an experienced anatomist. It may be remarked, however, that to succeed compleatly in this preparation, there should be, by preliminary incisions, performed according to art, certain foramina or apertures made, by which the saline solution may penetrate easily and readily through the texture of all the parts; and when it is intended to give freshness and the appearance of life to the cadaver, it will be requisite, previously, to fill the vessels and cellular tissues with a solution of coloured gelatine or jelly. There should be, likewise, placed in the ocular orbits, eyes of enamel, proportioned to the age and habitual condition of the subject. After these preparatory processes, the cadaver should be plunged in the saline solution of sur-oxygenated muriat of mercury, and be kept there longer or shorter, according to its bulk or magnitude; after which it should be taken out to let it dry slowly, and thus form a sort of mummy as durable as those of Egypt, and which has, moreover, the advantage of preserving the characters and essential traits of physiognomy.

During the two years since which Cit. Chaussier compiled this Memoir, he has continued his experiments, and made an application of his method to different objects; he has, consequently, found that the solution of sur-oxygenated muriat of mercury, not only preserved animal substances from putrefaction, but likewise that it stopped the progress of it when commenced, and brought them back, in some degree, to their former state. He has made use of it, with the greatest success, to preserve wood, cartoons, peltry, &c. from the voracity of insects. It may be likewise employed in cabinets of natural history for the preservation of birds and small quadrupeds. For example, instead of following the usual method of impaling or stuffing birds of a moderate size, Cit. Chaussier contents himself with making an incision on the mediant line of the abdomen. He removes the viscera which are contained in it, as, likewise, those of the thorax, at the base of the cranium, through the duct or channel of the gullet, (an aperture very convenient to take away the encephalon) and after having made, under the skin in the thickest part of the thighs, different issues or apertures, he plunges the body in the saline solution, keeps it there for a longer or shorter time, and then takes it out; and when it has sufficiently drained, he fills the abdomen and the thorax with fine tow, sews up the incision that had been made, and gives to the body the attitude which it ought afterwards to preserve. Insects may be destroyed or driven away from animals that have been a long time prepared, by plunging them during a certain time in the saline solution.

